# Desultory Reading

**Published:** 1874-04

**Authors:** 


### DESULTORY READING.

Some distinguished writers have laid down a very simple principle for the guidance of ordinary readers. Read, they have said, good books, and good books alone. Be familiar with the great masters of thought, and preserve your mind from the trash of the circulating library. The motives which prompt the advice are only too palpable. In days when a large proportion of the population is more or less capable of reading, it is melancholy to see that the effect is in one respect the very reverse of what might have been hoped. The greatest writers, though they may have positively a larger audience, have relatively a smaller audience than ever. Their works are pushed aside by masses of ephemeral literature, and even when read they are read with little attention. The mind becomes demoralized by the habit of desultory and superficial study; and a man who reads at a gallop expects that Shakspeare will yield up his secret as easily as the last new novelist. The greatest men are distinguished from the little men in nothing more than this, that the tenth or twentieth reading of their books is more fruitful than the first; whereas a modern reader is far too impatient to give more than one audience to the most venerable of teachers. Nothing, therefore, is more natural than to denounce as a debilitating practice all study of inferior authors. Life is shorter than ever in proportion to what has to be crowded into it, and our minds are not larger. We should, therefore, lay down immovable regulations against the invasion of distracting influences. The time which we dawdle away over the valueless parts of newspapers would enable us to become familiar with the thoughts of the wisest and best of men. If a man had to choose whether a few months hence he would be familiar with the ins and outs of the Tichborne case, or have made a careful study of all the Greek dramatists, no reasonable being could hesitate. In one case he would simply have enjoyed a questionable amusement, which leaves no traces behind it; in the other his imagination would have been stored with a perpetual source of delight. Yet hardly anybody has sufficient foresight or resolution to sacrifice the temporary excitement in consideration of the permanent advantage. The case, indeed, is, up to a certain point, too plain to admit of argument. Everybody should have an inner circle of friends amongst books, to which none but the really great writers should be admitted. So far as reading is not a mere pastime, but a part of the systematic cultivation of the faculties, it is only valuable in proportion as it implies close and intimate knowledge. No poetry is really worth reading unless it is worth learning by heart. A man may say that he has read Shakspeare's sonnets if he has glanced through them as he glances through a leading article; but he has not read them in any profitable sense until they have fascinated his imagination and sunk into his memory. Really great books, in short, must be assimilated, and they scarcely begin to produce their true influence until we know them so well that actual reference becomes almost superfluous. It is clearly desirable that every man should have thoroughly absorbed some of the masterpieces of literature as a true believer absorbs a book of religious devotion. If the task could be accomplished only by the sacrifice of all inferior work per haps it would be desirable to make the sacrifice.—*Saturday Review.*

